# Metformin Ameliorates A*β* Pathology by Insulin-Degrading Enzyme in a Transgenic Mouse Model of Alzheimer's Disease

**DOI:** 10.1155/2020/2315106

**Published:** 2020-04-19

**Authors:** Xin-Yi Lu, Shun Huang, Qu-Bo Chen, Dapeng Zhang, Wanyan Li, Ran Ao, Feona Chung-Yin Leung, Zhimin Zhang, Jisheng Huang, Ying Tang, Shi-Jie Zhang

**Affiliations:** ^1^Biological Resource Center, The Second Affiliated Hospital of Guangzhou University of Chinese Medicine, Guangzhou, China; ^2^Department of Neurology, The Second Affiliated Hospital of Guangzhou University of Chinese Medicine, Guangzhou, China; ^3^Nanfang PET Center, Nanfang Hospital, Southern Medical University, Guangzhou, China; ^4^The First Affiliated Hospital of Guangzhou Medical University, Guangzhou, China; ^5^School of Chinese Medicine, LKS Faculty of Medicine, The University of Hong Kong, Hong Kong; ^6^Drug Non-Clinical Evaluation Center of Guangzhou Institute of Pharmaceutical Industry, Guangzhou General Pharmaceutical Research Institute Co. Ltd., Guangzhou, China; ^7^School of Basic Medical Sciences, Center for Post-Doctoral Studies of Southern Medical University, Guangzhou, China; ^8^Post-Doctoral Research Center of Guangzhou Pharmaceutical Holdings Ltd., Guangzhou, China; ^9^The First Affiliated Hospital of Guangzhou University of Chinese Medicine, Guangzhou University of Chinese Medicine, Guangzhou, China

## Abstract

Alzheimer's disease (AD) is the most common neurodegenerative disease. The accumulation of amyloid beta (A*β*) is the main pathology of AD. Metformin, a well-known antidiabetic drug, has been reported to have AD-protective effect. However, the mechanism is still unclear. In this study, we tried to figure out whether metformin could activate insulin-degrading enzyme (IDE) to ameliorate A*β*-induced pathology. Morris water maze and Y-maze results indicated that metformin could improve the learning and memory ability in APP^swe^/PS1^dE9^ (APP/PS1) transgenic mice. ^18^F-FDG PET-CT result showed that metformin could ameliorate the neural dysfunction in APP/PS1 transgenic mice. PCR analysis showed that metformin could effectively improve the mRNA expression level of nerve and synapse-related genes (*Syp*, *Ngf*, and *Bdnf*) in the brain. Metformin decreased oxidative stress (malondialdehyde and superoxide dismutase) and neuroinflammation (IL-1*β* and IL-6) in APP/PS1 mice. In addition, metformin obviously reduced the A*β* level in the brain of APP/PS1 mice. Metformin did not affect the enzyme activities and mRNA expression levels of A*β*-related secretases (*ADAM10*, *BACE1*, and *PS1*). Meanwhile, metformin also did not affect the mRNA expression levels of A*β*-related transporters (*LRP1* and *RAGE*). Metformin increased the protein levels of p-AMPK and IDE in the brain of APP/PS1 mice, which might be the key mechanism of metformin on AD. In conclusion, the well-known antidiabetic drug, metformin, could be a promising drug for AD treatment.

## 1. Introduction

Alzheimer's disease (AD), a progressive neurodegenerative disease with a high incidence rate in this century, has cognitive and functional ability decline with the disease progress. AD is mainly characterized by the accumulation of amyloid beta (A*β*) plaques and neurofibrillary tangles (NFTs) in the brain [1–3]. Accumulation of A*β*, which is generated from the amyloid precursor protein (APP), is the main hallmark of AD [4]. Thus, targeting A*β* is leading to a potential therapeutic strategy [5]. However, a series of clinical trials, such as inhibitors of *β*-secretase (BACE1) or *γ*-secretase (PS1), have failed [6, 7]. Promoting the degradation or clearance of A*β* is considered as an alternative therapeutic strategy [8].

Diabetes mellitus (DM) is also associated with a higher risk of AD [9, 10], in which insulin deficiency or insulin resistance may be responsible. Metformin, as a major antidiabetic drug, has been demonstrated to reduce *β*-secretase activity, promote phospho-thr-231-tau degradation, and influence mitochondrial function [10–14]. Recently, some molecules, which are widely studied in diabetes, have been proved to affect A*β* clearance, including insulin-degrading enzyme (IDE), neprilysin (NEP), receptor for advanced glycation end products (RAGE), and matrix metalloproteinases (MMPs) [15–19]. Among these molecules, IDE plays an important role in both diabetes and AD. Loss-of-function mutation in the IDE gene can lead to impaired degradation of A*β* [20]. Colocalization and codeposition of IDE with A*β* plaques can be found in the AD brain. Inhibition of IDE in the brain is identified as one of the major factors involved in the crosstalk between DM and AD [21]. However, whether metformin could protect against AD through the IDE pathway is still unknown.

APP^swe^/PS1^dE9^ (APP/PS1) double transgenic mice were used for the study. The APP/PS1 mouse exhibits the A*β* plaque formation and memory impairment, which is similar to clinical phenotype [22], including memory deficits, anxiety, hyperactivity, and social interaction impairment. Metformin, an experimental therapy, was used to explore the hypothesis that metformin protects against AD via IDE signaling in APP/PS1 mice.

## 2. Material and Methods

### 2.1. Materials

Metformin and Thioflavin T (ThT) reagent were purchased from Sigma-Aldrich (Saint Louis, MO, USA). A BCA protein assay kit and superoxide dismutase (SOD) assay kit were obtained from Beyotime Biotechnology. The malondialdehyde (MDA) Assay Kit (TBA method) was purchased from Nanjing Jiancheng Bioengineering Institute (Nanjing, China). qPCR reagents and ECL kit were purchased from Invitrogen. All the antibodies were obtained from Cell Signaling Technology, Inc. (Danvers, MA, USA).

### 2.2. Animals and Treatments

The 7-month-old male APP/PS1 double transgenic mice and wild-type mice (C57BL/6) were obtained from the Model Animal Research Institute of Nanjing University (Nanjing, China) and maintained at the laboratory animal center of Guangzhou Medical University under the standard housing conditions with free access to food and water. The animal management was approved by the Institutional Animal Care and Use Committee of Guangzhou Medical University (SCXK2019-0013). All mice were randomly divided into three groups: wild type (WT, *n* = 15), APP/PS1 (*n* = 15), and APP/PS1+metformin (200 mg/kg/day, *n* = 15) [23, 24]. After oral drug administration for 8 weeks, mice were performed behavioral tests and subsequently sacrificed for the collection of brains.

### 2.3. Morris Water Maze Test

The Morris water maze test was performed to evaluate the spatial memory performance. The opaque platform with a diameter of 10 cm was positioned 1 cm beneath the water surface. The duration of training and testing session was 60 s. In the training session, mice completed four trials daily with at least 20 minutes of interval between two trials for six consecutive days. Each mouse was released into the water by facing the wall in one of the four quadrants. If the mouse failed to reach the platform within 60 s, it would be directed to the platform and stay there for 15 s. In the testing session, the platform was removed from the pool and the mice were allowed to search for the platform for 60 s. A computerized video imaging analysis system (Feidi, Guangzhou) was used to record and analyze the swimming paths in the maze.

### 2.4. Y-Maze Test

The Y-maze test was performed to evaluate the working memory performance. The apparatus consisted of three arms (one start arm and two goal arms) of 30 × 10 × 20 cm connected by an intersection. The duration of training and testing session was 2 min. Before the training and testing sessions, the body weight of mice would reduce to 90% by food restriction. In the training session, mice completed 10 trials daily with at least 20 min interval between two trials for four consecutive days. Prior to the reward alternation testing session, each mouse would consume the food which was filled at the end of the arm for 4-6 times to habituate to the maze. The choice for each goal arm should be given equally, and the percentage of correct choices would be calculated to analysis.

### 2.5. ^18^F-FDG PET Imaging

After 8 weeks of metformin administration, the microPET-CT was used to evaluate brain glucose uptake. ^18^F-Fluordeoxyglucose (^18^F-FDG) was intraperitoneally injected into animals. The mice were scanned by using a Focus 220 microPET scanner (Siemens Medical Solutions USA, Inc., Knoxville, TN, USA). Dynamic scans were conducted for 1 h. PET images were reconstructed using the microPET-CT manager (Siemens Medical Solutions USA, Inc.). To evaluate relative glucose metabolism, the ratio of the SUV standardized uptake value was obtained by dividing the SUV of each region with the SUV of the whole brain.

### 2.6. ELISA

Brain tissues were homogenized with saline, containing a cocktail of protease inhibitors. Samples were centrifuged by 12000 × *g* for 15 min. The supernatants were measured for IL-1*β*, IL-6 (Thermo Fisher Scientific), A*β*1-40, A*β*1-42 (Invitrogen), and *α*-, *β*-, and *γ*-secretases (R&D Systems, USA) by using the ELISA kits according to the manufacturer's proposals.

### 2.7. ThT Staining

The sections were washed three times in PBS at room temperature and then incubated with a ThT reagent (50 *μ*M) for 30 min. After washing three times in PBS, the sections were captured by a fluorescence microscope (Leica).

### 2.8. Measurement of SOD and MDA

The brain tissues were homogenized and centrifuged by 12000 × *g* for 15 min. The supernatants were used to test SOD activity and MDA level according to the kit instructions (Nanjing Jiancheng Bioengineering Institute).

### 2.9. qPCR

Total RNA from brain tissues were extracted using a TRIzol reagent. Reverse transcription was treated with an ExScript RT Reagent Kit (Invitrogen). Real-time PCR analysis was undertaken using SYBR Premix Ex Taq (Invitrogen). The transcriptions were investigated for several target genes, including *synaptophysin* (*Syp*): For 5′-GTGCTGCAATGGGTCTTCG-3′ and Rev 5′-CCGTGGCCAGAAAGTCCAG-3′; *nerve growth factor* (*Ngf*): For 5′-CAAGGACGCAGCTTTCTATACTG-3′ and Rev 5′-CTTCAGGGACAGAGTCTCCTTCT-3′; *brain-derived neurotrophic factor* (*Bdnf*): For 5′-TACTTCGGTTGCATGAAGGCG-3′ and Rev 5′-GTCAGACCTCTCGAACCTGCC-3′; *a disintegrin and metalloproteinase domain-containing protein 10* (*ADAM10*): For 5′-TTCTCCCTCCGGATCGATGT-3′ and Rev 5′-ATACTGACCTCCCATCCCCG-3′; *beta-secretase 1* (*BACE1*): For 5′-ACTTTACACTCTGTTCTGGGTGG-3′ and Rev 5′-ACCACAAAGCCTGGCAATCTC-3′; *presenilin 1* (*PS1*): For 5′-AATGACGACAACGGTGAGGG-3′ and Rev 5′-CCAGATTAGGTGCTTCCCCG-3′; *low-density lipoprotein receptor-related protein 1* (*LRP1*): For 5′-GGACCACCATCGTGGAAA-3′ and Rev 5′-TCCCAGCCACGGTGATAG-3′; *receptor for advanced glycation end products* (*RAGE*): For 5′-GGACCCTTAGCTGGCACTTAGA-3′ and Rev 5′-GAGTCCCGTCTCAGGGTGTCT-3′; and *β*-actin: For 5′-AGAGCTACGAGCTGCCTGAC-3′ and Rev 5′-AGCACTGTGTTGGCGTACAG-3′.

### 2.10. Western Blotting

The fresh brain tissues were homogenated with RIPA buffer containing 1% phenylmethanesulfonyl fluoride and phosphatase inhibitors. The samples was separated by sodium dodecyl sulfate polyacrylamide gel electrophoresis (SDS-PAGE) and transferred onto PVDF membranes. The blotting membranes were blocked with 5% BSA. The membranes were incubated at 4°C overnight with the following primary antibodies: anti-p-AMP-activated protein kinase (AMPK), anti-AMPK, anti-IDE, anti-NEP, and anti-*β*-actin. All the antibodies were purchased from CST. The membranes were incubated with a secondary antibody at room temperature. The membranes were covered with mixed liquid from the ECL Chemiluminescent Substrate Reagent Kit. The bands were scanned by a luminescent image analyzer (Invitrogen).

### 2.11. Statistical Analysis

All statistical analyses were conducted by SPSS 20.0 software. One-way analysis of variance (ANOVA) and Student *t*-test were used to analyze. Data were expressed as means ± SEM. *p* < 0.05 was considered significant.

## 3. Results

### 3.1. Metformin Ameliorates Learning and Memory Dysfunctions in APP/PS1 Mice

**Figure 1:**
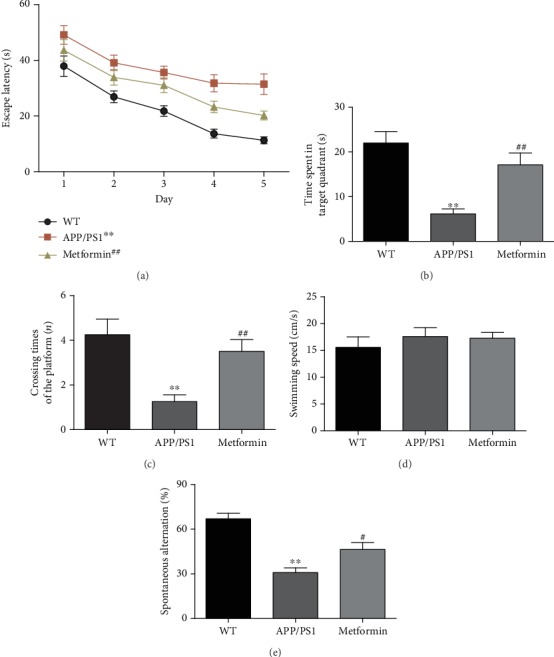
Metformin improves learning and memory impairment in APP/PS1 mice. (a) Escape latency of the five-day Morris water maze. (b) Time spent in the target quadrant in the Morris water maze. (c) Crossing times of the target platform in the Morris water maze. (d) Swimming speed in the Morris water maze. (e) Percentage of spontaneous alternation of Y-maze. Data represent the mean ± SEM (*n* = 15 per group). ^∗^*p* < 0.05, ^∗∗^*p* < 0.01, and ^∗∗∗^*p* < 0.001 vs. WT; ^#^*p* < 0.05, ^##^*p* < 0.01, and ^###^*p* < 0.001 vs. APP/PS1.

To prove the therapeutic effect of metformin on APP/PS1 mice, the Morris water maze and Y-maze tests were used to assess the cognitive function. Results showed that APP/PS1 mice suffered an obvious decline in cognitive function. Metformin could significantly improve escape latency, increase the crossing times, and shorten the time of finding the platform (Figures 1(a)–1(c)). Swimming speed differences among the three groups did not have any statistical significance (Figure 1(d)). In the Y-maze test, APP/PS1 mice showed a significant downward trend in spontaneous alternation, compared to the WT group. After metformin treatment, the mice exhibited better performance than the APP/PS1 group (Figure 1(e)).

### 3.2. Metformin Improves Brain Function in APP/PS1 Mice

**Figure 2:**
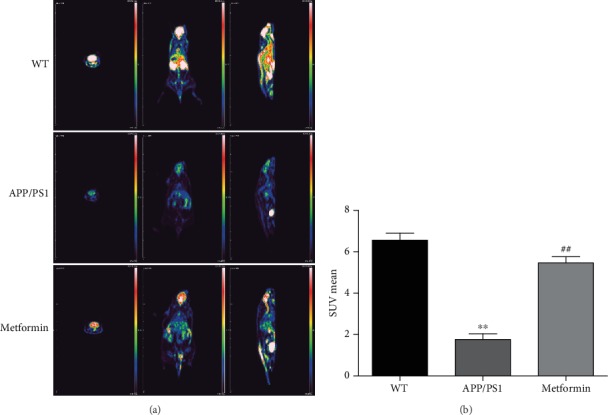
Metformin improves glucose metabolism in APP/PS1 mice. (a) PET-CT images. (b) ^18^F-FDG uptake of mice brains. Data represent the mean ± SEM (*n* = 3 per group). ^∗^*p* < 0.05, ^∗∗^*p* < 0.01, and ^∗∗∗^*p* < 0.001 vs. WT; ^#^*p* < 0.05, ^##^*p* < 0.01, and ^###^*p* < 0.001 vs. APP/PS1.

**Figure 3:**
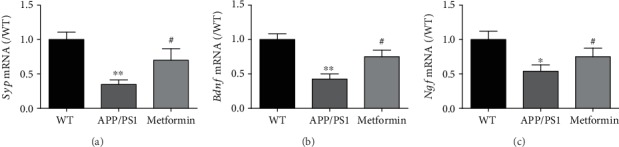
Metformin improves neurotrophic factors in APP/PS1 mice. The mRNA levels of (a) *Syp*, (b) *Bdnf*, and (c) *Ngf* in the APP/PS1 mice. Data represent the mean ± SEM (*n* = 6 per group). ^∗^*p* < 0.05, ^∗∗^*p* < 0.01, and ^∗∗∗^*p* < 0.001 vs. WT; ^#^*p* < 0.05, ^##^*p* < 0.01, and ^###^*p* < 0.001 vs. APP/PS1.

Decreased glucose metabolism is the characteristic symptom of AD. ^18^F-FDG-PET was used to evaluate the cerebral metabolism. As shown in Figure 2, microPET-CT imaging suggested that ^18^F-FDG uptake intensity was sharply decreased in the brain of APP/PS1 mice. Metformin remarkably increased ^18^F-FDG uptake in the brain of APP/PS1 mice. In addition, the mRNA expression levels of neurotrophic factors (*Bdnf* and *Ngf)* and synaptic factor (*Syp*) were significantly reduced in APP/PS1 mice (Figures 3(a)–3(c)). Metformin significantly improved the mRNA expression levels of neurotrophic factors and synapse-related proteins.

### 3.3. Metformin Reduces Oxidative Stress and Inflammation in the Brain of APP/PS1 Mice

**Figure 4:**
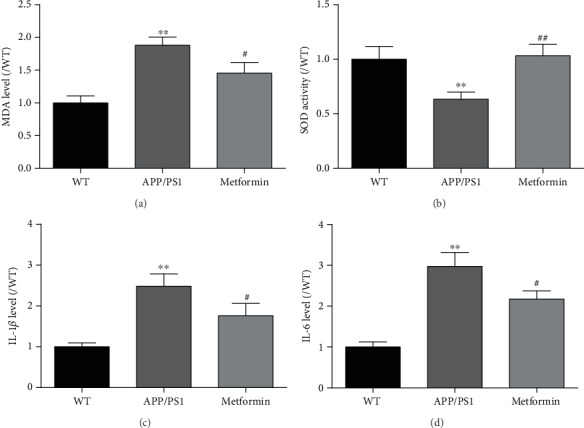
Metformin ameliorates oxidative stress and neuroinflammation in APP/PS1 mice. The level of (a) MDA and the activity of (b) SOD in the brain of APP/PS1 mice. The levels of (a) IL-1*β* and (b) IL-6 in the brain of APP/PS1 mice. Experimental values were expressed as the mean ± SEM (*n* = 6 per group). ^∗^*p* < 0.05, ^∗∗^*p* < 0.01, and ^∗∗∗^*p* < 0.001 vs. WT; ^#^*p* < 0.05, ^##^*p* < 0.01, and ^###^*p* < 0.001 vs. APP/PS1.

Neural oxidative stress and inflammation are the key pathologies of AD [25]. In the brain of APP/PS1 mice, the level of MDA was increased, and the activity of SOD was reduced (Figures 4(a) and 4(b)). Metformin significantly relieved the oxidative stress status. In addition, the levels of inflammatory markers, IL-1*β* and IL-6, were significantly increased in the brain of APP/PS1 mice (Figures 4(c) and 4(d)). Metformin reduced the levels of IL-1*β* and IL-6. These data suggested that metformin could inhibit oxidative stress and inflammation in APP/PS1 mice.

### 3.4. Metformin Reduces A*β* Accumulation in the Brain of APP/PS1 Mice

**Figure 5:**
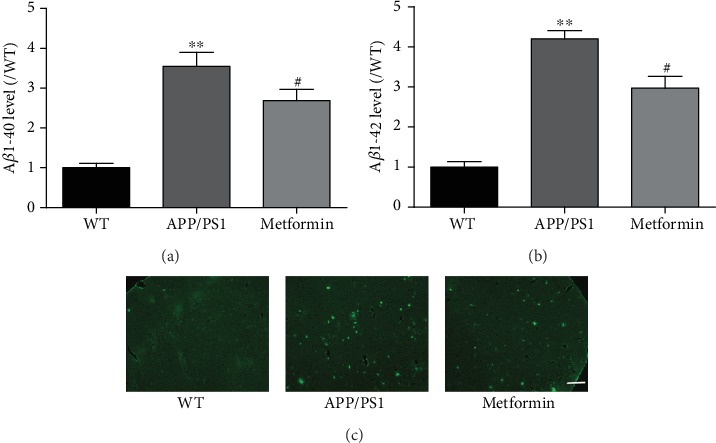
Metformin decreases A*β* levels in APP/PS1 mice. The levels of (a) A*β*1-40 and (b) A*β*1-42 in the brain of APP/PS1 mice. ThT staining of the brain slides in APP/PS1 mice. Experimental values were expressed as the mean ± SEM (*n* = 6 per group). ^∗^*p* < 0.05, ^∗∗^*p* < 0.01, and ^∗∗∗^*p* < 0.001 vs. WT; ^#^*p* < 0.05, ^##^*p* < 0.01, and ^###^*p* < 0.001 vs. APP/PS1. Bar: 100 *μ*m.

The accumulation of A*β* is the main pathological feature of AD. The brain of APP/PS1 mice is overloaded with A*β*. Thus, A*β* levels were further studied. ELISA results indicated that metformin effectively reduced the levels of A*β*1-40 and A*β*1-42 in the brain of APP/PS1 mice (Figures 5(a) and 5(b)). ThT staining results further confirmed that metformin ameliorated A*β* accumulation in the brain of APP/PS1 mice (Figure 5(c)). These were strong evidences that metformin could reduce A*β* accumulation in APP/PS1 mice.

### 3.5. Metformin Activates AMPK and Increases IDE in the Brain of APP/PS1 Mice

**Figure 6:**
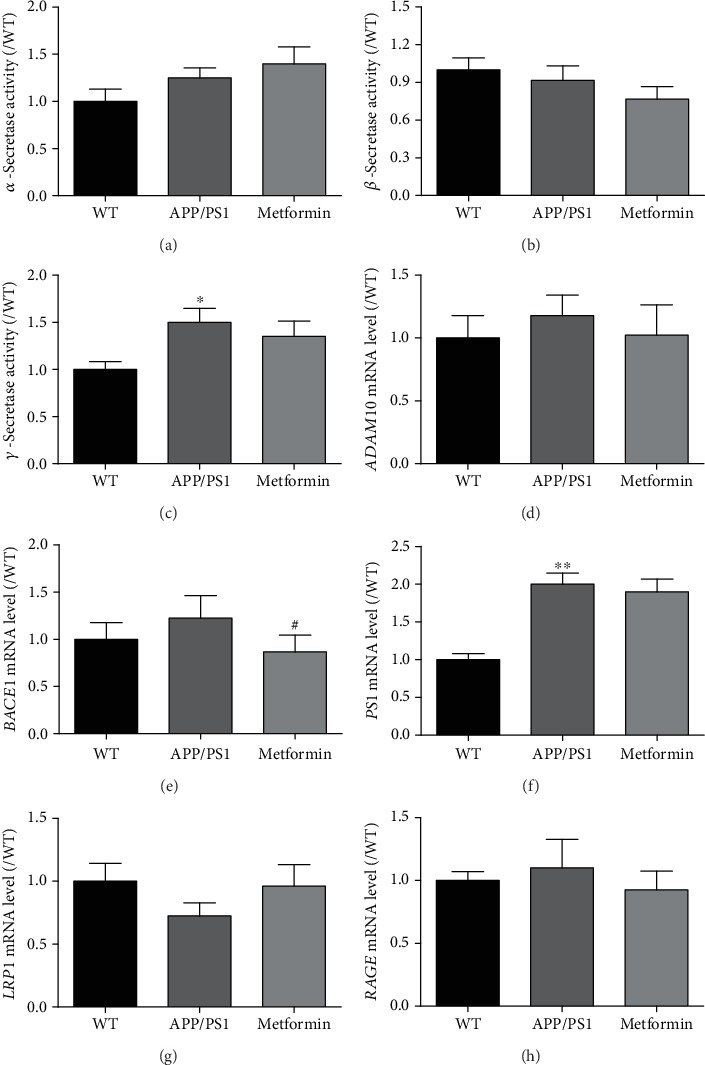
Metformin has no effect on A*β* production and transportation-related genes in APP/PS1 mice. The activities of (a) *α*-, (b) *β*-, and (c) *γ*-secretases. The mRNA expressions of (d) *ADAM10*, (e) *BACE1*, (f) *PS1*, (g) *LRP1*, and (h) *RAGE*. Experimental values were expressed as the mean ± SEM (*n* = 6 per group). ^∗^*p* < 0.05, ^∗∗^*p* < 0.01, and ^∗∗∗^*p* < 0.001 vs. WT; ^#^*p* < 0.05, ^##^*p* < 0.01, and ^###^*p* < 0.001 vs. APP/PS1.

**Figure 7:**
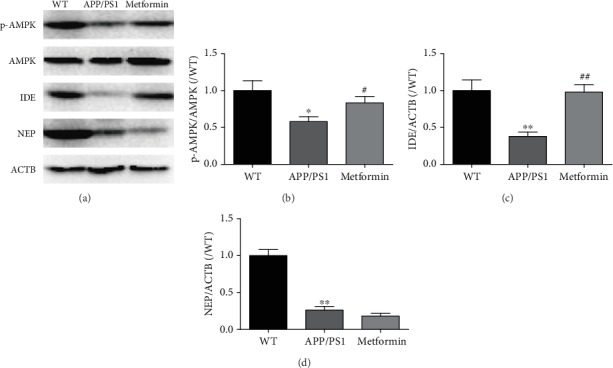
Metformin activates AMPK and increases IDE in the brain of APP/PS1 mice. (a) The representative bands of p-AMPK, AMPK, IDE, NEP, and ACTB. Western blot analysis: (b) p-AMPK/AMPK, (c) IDE/ACTB, and (d) NEP/ACTB. Experimental values were expressed as the mean ± SEM (*n* = 3 per group). ^∗^*p* < 0.05, ^∗∗^*p* < 0.01, and ^∗∗∗^*p* < 0.001 vs. WT; ^#^*p* < 0.05, ^##^*p* < 0.01, and ^###^*p* < 0.001 vs. APP/PS1.

We next studied how metformin influences A*β* metabolism. Sequential cleavage of the APP by *β*- and *γ*-secretases can produce A*β* [26]. Thus, we firstly detected these secretases. ELISA results showed that metformin had no effect on *α*-, *β*-, or *γ*-secretase (Figures 6(a)–6(c)). The mRNA expression levels of *ADAM10*, *BACE1*, and *PS1* were also tested. Metformin did not affect these gene expression levels except for a slight decrease of *BACE1* (Figures 6(d)–6(f)). We next detected the A*β* transportation-related gene. Results showed that metformin also had no effect on the mRNA expression levels of *LRP1* and *RAGE* (Figures 6(g) and 6(h)). In addition, IDE and NEP, as the degrading enzymes of A*β*, are other key aspects of the process of A*β* clearance. We assessed the protein expression levels of IDE and NEP in the brain of APP/PS1 mice (Figure 7). The IDE and NEP expression levels were significantly decreased in APP/PS1 mice compared to wild-type mice. Metformin significantly increased the expression level of IDE, but not NEP, in the APP/PS1 mice. Previous studies have reported that the AMPK pathway is involved in metformin effect [24, 27–30]. Our results verified this phenomenon. Metformin significantly increased the protein expression level of p-AMPK. These data suggested that the IDE signaling pathway might participate in the neuroprotective effect of metformin.

## 4. Discussion

In this study, we verified that the antidiabetic drug, metformin, could effectively ameliorate AD symptom in APP/PS1 double transgenic mice. After 8 weeks' treatment, we found that metformin could relieve learning and memory dysfunction and improve brain function. Meanwhile, metformin signally inhibited oxidative stress and neuroinflammation. Furthermore, metformin activates AMPK and increases IDE in the brain of APP/PS1 mice, which might be the key neuroprotective mechanism of metformin.

A*β* accumulation is the main pathology of AD, which can cause the cascade reaction and induce neural apoptosis [31]. Studies have shown that metformin is beneficial for AD patients [10–12]. In this experiment, we further investigated the neuroprotective mechanism of metformin on APP/PS1 mice. We did the preliminary experiments: different dosages of metformin (25, 50, 100, and 200 mg/kg/day) were given to APP/PS1 mice. Morris water maze test results indicated that metformin (200 mg/kg/day) was the best dosage (data not shown), which was consistent with previous studies [23, 24]. Behavioral studies (Morris water maze and Y-maze) confirmed that metformin could significantly improve learning and memory in APP/PS1 mice, which was consistent with previous studies. In addition, metformin improved cerebral metabolism and brain function and reduced the level of A*β*.

A*β* accumulation exacerbates oxidative damage and inflammation. A*β* can increase the synthesis of the superoxide anion, reduce the activity of catalase and SOD, activate MDA production, and finally produce reactive oxygen species [30]. In our findings, we found that the contents of MDA were significantly increased and SOD activity was significantly decreased in APP/PS1 mice. After metformin treatment, oxidative stress was relieved. A*β* accumulation can increase the levels of IL-1*β* and IL-6, the proinflammatory factors, in APP/PS1 mice [32, 33]. Neuroinflammation can also exacerbate AD pathology [25]. In this study, metformin reduced the levels of IL-1*β* and IL-6 in APP/PS1 mice.

A*β* production is strongly linked with *α*-, *β*-, and *γ*-secretases [26]. When APP is proteolytically processed by *β*- and *γ*-secretases, A*β* production is increased. When the activity of *α*-secretase is increased, A*β* production is inhibited. A*β* transportation is another way of A*β* metabolism. RAGE and LRP1 are the two main molecules, which participate in A*β* transportation [34]. In this study, both ELISA and qPCR results showed that metformin had little influence on *α*-, *β*-, and *γ*-secretases and RAGE and LRP1, except for a small reduction in *BACE1* expression, which was consistent with a previous study [11]. These results indicated that except A*β* production and transportation, other signaling pathways might also be involved in the effect of metformin.

AMPK is a crucial factor in the regulation of intracellular systems [30]. AMPK activation can enhance anti-inflammatory effect, which might be regulated by AMPK/mTOR and AMPK/NF-*κ*B signaling pathways [24, 27–30]. Metformin was supposed to have a potential pharmacological effect, due to AMPK activation [35]. In some studies, AMPK/SIRT1 takes part in the nonamyloidogenic pathway to improve AD [36]. In this study, metformin obviously activated AMPK in the brain of APP/PS1 mice. In regard to A*β* clearance, IDE and NEP are the main cellular degrading enzymes of A*β*. Early findings showed that IDE could regulate A*β* and insulin levels in vivo [37]. The major locations of IDE are the cytosol, mitochondria, and peroxisomes [38]. IDE is specific toward *β*-structure-forming substrates of toxic oligomers (A*β*) [39]. The activity of IDE in the brain decreases with age and during early stages of AD. Overexpression of IDE in transgenic mice can prevent amyloid plaque formation [40]. Inhibition of IDE is identified as one of the crosstalk between T2D and AD [21]. In our findings, the protein expression levels of IDE and NEP were significantly decreased in APP/PS1 mice. Metformin effectively increased the protein level of IDE. These data indicated that the IDE pathway might also participate in the neuroprotective effect of metformin.

In conclusion, we provided the evidence that metformin had beneficial effects by reducing the A*β* level through the IDE pathway in APP/PS1 mice. In addition, metformin could decrease inflammation and oxidative stress. However, further studies are still needed. Metformin might offer a new promising avenue in AD treatment.

## Data Availability

The data used to support the findings of this study are available from the corresponding author upon request.
